# C-reactive protein can upregulate VEGF expression to promote ADSC-induced angiogenesis by activating HIF-1α via CD64/PI3k/Akt and MAPK/ERK signaling pathways

**DOI:** 10.1186/s13287-016-0377-1

**Published:** 2016-08-16

**Authors:** JiaYuan Chen, ZhenJie Gu, MaoXiong Wu, Ying Yang, JianHua Zhang, JingSong Ou, ZhiYi Zuo, JingFeng Wang, YangXin Chen

**Affiliations:** 1 Department of Cardiology, Sun Yat-sen Memorial Hospital, Sun Yat-sen University, Guangzhou, 510120 People’s Republic of China; 2 Guangdong Province Key Laboratory of Arrhythmia and Electrophysiology, Guangzhou, 510120 People’s Republic of China; 3 Laboratory of RNA and Major Disease of Brain and Heart, Sun Yat-sen Memorial Hospital, Sun Yat-sen University, Guangzhou, 510120 People’s Republic of China; 4 Division of Cardiac Surgery, The First Affiliated Hospital, Sun Yat-sen University, Guangzhou, 510080 People’s Republic of China; 5 Guangdong Province Engineering Laboratory for Diagnosis and Treatment of Vascular Diseases, Guangzhou, 510080 People’s Republic of China; 6 Department of Anesthesiology, University of Virginia, Health Science Center, Charlottesville, VA USA

**Keywords:** C-reactive protein, Angiogenesis, Adipose-deprived stem cell, Vascular endothelial growth factor, Hypoxia-inducible factor-1α

## Abstract

**Background:**

Proliferation of the vasa vasorum has been implicated in the pathogenesis of atherosclerosis, and the vasa vasorum is closely associated with resident stem cells within the vasculature. C-reactive protein (CRP) is positively correlated with cardiovascular disease risk, and our previous study demonstrated that it induces inflammatory reactions of perivascular adipose tissue by targeting adipocytes.

**Methods:**

Here we investigated whether CRP affected the proliferation and proangiogenic paracrine activity of adipose-derived stem cells (ADSCs), which may contribute to vasa vasorum angiogenesis.

**Results:**

We found that CRP did not affect ADSC apoptosis, cell cycle, or proliferation but did increase their migration by activating the PI3K/Akt pathway. Our results demonstrated that CRP can upregulate vascular endothelial growth factor-A (VEGF-A) expression by activating hypoxia inducible factor-1α (HIF-1α) in ADSCs, which significantly increased tube formation on Matrigel and functional vessels in the Matrigel plug angiogenesis assay. The inhibition of CRP-activated phosphorylation of ERK and Akt can suppress CRP-stimulated HIF-1α activation and VEGF-A expression. CRP can also stimulate proteolytic activity of matrix metalloproteinase-2 in ADSCs. Furthermore, CRP binds activating CD64 on ADSCs, rather than CD16/32.

**Conclusion:**

Our findings implicate that CRP might play a role in vasa vasorum growth by activating the proangiogenic activity of ADSCs.

**Electronic supplementary material:**

The online version of this article (doi:10.1186/s13287-016-0377-1) contains supplementary material, which is available to authorized users.

## Background

C-reactive protein (CRP), an acute-phase protein and a member of the pentraxin family, members of which are characterized by a cyclic pentameric structure, exhibits Ca^2+^-dependent binding to ligands and binds to the membrane of injured cells as well as the membrane and nuclear components of necrotic and apoptotic cells [1]. Baseline circulating concentrations of CRP are significantly associated with cardiovascular disease risk in the general population [2]. Recent clinical trials and basic research demonstrated that CRP could be a proatherogenic factor for atherosclerosis [3], whereas in-vitro experiments reported that CRP preparations had been contaminated by bacterial products or other contaminated preparations [4, 5], and a recent study showed that no proinflammatory cytokines or acute phase proteins were detected after purified CRP from pooled normal human donor plasma was infused into seven healthy adult human volunteers [6]. All evidence indicates that the functions of CRP still remain controversial as a biomarker or mediator.

Research into the pathogenesis of atherosclerosis has focused historically on the intima and media. However, recent studies demonstrated that adventitial vasa vasorum angiogenesis and periadventitial adipose tissue inflammation play an important role in the development of coronary atherosclerosis, known as the “outside-in” phenomenon [7, 8]. Manka et al*.* [9] reported that the transplantation of perivascular adipose tissue (PVAT) from donor mice to the carotid arteries can promote vasa vasorum neovascularization in the adventitia, indicating that PVAT inflammation played a role in adventitia vasa vasorum angiogenesis. A certain amount of mesenchymal stem cells within adipose tissue, including PVAT [10], were closely associated with new vessel angiogenesis [11]. Stem cells are thought to be quiescent or to cycle slowly under normal circumstances, and the biological function of stem cells is activated by microenvironmental reactions such as inflammation, hypoxia, and oxidative stress. Whether PVAT inflammation could promote mesenchymal stem cell-induced vasa vasorum angiogenesis is not clearly understood.

PVAT inflammation is often accompanied by increased circulating CRPs. Because we know that the imbalance of adiponectin and leptin is the main cause of adipose tissue inflammation, increased leptin is able to further promote CRP production from hepatocytes and endothelial cells [12]. It is therefore interesting to investigate the role of CRP in PVAT inflammation. Our previous study showed that CRP could activate inflammatory reactions within PVAT by stimulating cultured adipocytes to release tumor necrosis factor alpha, interleukin-6, and monocyte chemoattractant protein-1 (MCP-1) and enhancing macrophage infiltration [13], indicating that CRP might act as a mediator in PVAT inflammation.

On the other hand, CRP could be a potent activator of angiogenesis. Recent studies showed that the inhibition of endothelial cell angiogenesis and increased apoptosis by CRP may be attributed to the presence of sodium azide in CRP preparations. Slevin et al. [14] reported that CRP is associated with the formation of immature microvessels in vivo, which is significantly expressed by stroke neovessels. In vitro, CRP can increase vascular endothelial growth factor (VEGF)-A expression in bovine aortic endothelial cells, human coronary artery endothelial cells, and monocytes, which was due to CRP itself but not the effects of sodium azide and lipopolysaccharide (LPS) contamination [15–17]. However, whether CRP can also promote the proliferation and proangiogenic paracrine activity of adipose-derived stem cells (ADSCs) as an angiogenic factor, which contribute to PVAT inflammation-related vasa vasorum angiogenesis, is still poorly defined. We hypothesized that human CRP promotes ADSC-induced angiogenesis in the setting of atherosclerosis. To test this hypothesis, we investigated the role of CRP on the proliferation, migration, and paracrine proangiogenic activity of ADSCs and identified the signaling pathways and the molecular mechanisms in vitro.

## Methods

### Mouse ADSC isolation and cell culture

Primary mouse ADSCs from mouse adipose tissue were isolated and cultured as described previously with minor modifications [18]. The fatty tissue around the inguinal region of male C57/BL6 mice, 3–4 weeks old, was separated. After the removal of visible blood vessels, lymph nodes, and fascia, the tissue was finely minced with scissors and digested with collagenase type I (1.25 % w/v) for 60 min at 37 °C with gentle shaking. After collagenase neutralization, the floating adipocytes were separated by centrifugation at 1200 rpm for 5 min. The resulting pellet was resuspended and the cells were plated in tissue culture flasks in Dulbecco’s modified Eagle’s medium with low glucose (DMEM; Gibco, Thermo Fisher Scientific, Inc., Waltham, MA, USA) supplemented with 10 % fetal bovine serum (FBS; Gibco, Thermo Fisher Scientific, Inc.), 100 U/ml penicillin and 0.1 mg/ml streptomycin (both from Thermo Fisher Scientific, Inc., Waltham, MA, USA), VEGF 10 ng/ml, basic fibroblast growth factor (FGF) 10 ng/ml, and alpha-FGF 10 ng/ml (Sigma-Aldrich, St. Louis, MO, USA) at 37 °C in a 5 % CO_2_ humidified atmosphere.

### Flow cytometry analysis

Cell apoptosis was detected by an Annexin V-FITC apoptosis detection kit according to the manufacturer’s instructions. The cells were incubated with or without the addition of various concentrations of recombinant human CRP (free of sodium azide; Sino Biological Inc., Beijing, China) for different times and then harvested and rinsed in cold phosphate-buffered saline (PBS). The fraction of apoptotic cells was determined by cell staining in Annexin-V binding buffer with FITC-conjugated Annexin-V and propidium iodide (PI; Sigma-Aldrich). After 15 min of incubation in the dark at room temperature, the samples were analyzed by flow cytometry (LSRII FACS; BD Bioscience, Franklin Lakes, NJ, USA). Apoptotic cells were identified as Annexin-V-positive cells.

For the cell cycle analysis, the cells were trypsinized and fixed in 75 % ethanol for 60 min on ice and stained with PI and Hoechst 33342 (5 μg/ml; Thermo Fisher Scientific, Inc.) in PBS for 30 min. Equal numbers of cells were assessed for ADSCs by flow cytometry analysis.

### Cell proliferation assay

ADSCs were seeded in 96-well plates at a density of 4 × 10^3^ cells/well. After 24, 48, and 72 hours, the medium was removed and cells were counted using a Cell Counting Kit-8 (CCK-8; Dojindo, Rockville, MD, USA). Cells were treated with 10 % CCK-8 solution for 4 hours at 37 °C in a humidified 5 % CO_2_ incubator and the absorbance was measured at 450 nm by a microplate reader.

### Cell migration assay

ADSCs were seeded on the upper site of 8.0 μm transwell membrane plates (Corning, Inc., NY, USA) at a density of 5 × 10^4^ cells per well after serum starvation for 12 hours. CRP (25 μg/ml) or plus inhibitors (PD098059, 10 μM; LY294002, 5 μM) in DMEM were introduced on the lower site of transwell membrane plates for 12–24 hours. Migrated cells remaining in the transwell membrane were fixed and then stained using 10 % crystal-violet (Sigma-Aldrich), and cells in the membrane were counted by light microscopy.

### Inhibitor and block antibody treatment

After reaching 80 % confluence, ADSCs were seeded in six-well plates (5 × 10^5^ cells/well). The cells were treated with the following reagents for 24 hours: (a) ADSC basal medium as a control; (b) stimulant CRP alone; (c) stimulant plus inhibitors or block antibodies, including ERK inhibitor (PD098059, 10 μM), PI3K inhibitor (LY294002, 5 μM), and nuclear factor-kappa beta (NF-kB) inhibitor (BAY-11-7082, 5 μM) (all from Cell Signaling Technology, Danvers, MA, USA), or anti-CD16 (2 μg/ml; R&D Systems, Inc., Madison, WI, USA), anti-CD16/32 (1 μg/ml; Abcam Inc., Cambridge, UK), and anti-CD64 (1:100, 3 μg/ml; R&D Systems Inc.); or (d) inhibitors and block antibodies alone. Doses of the inhibitors or block antibodies were determined according to previous laboratory characterization and published data. Supernatants and cell extractions were collected 24 hours after treatment.

### In-vitro tube formation assay

Tube formation on Matrigel was performed as described previously [19]. A total of 50 μl of chilled Matrigel (BD Bioscience) was added to a 96-well plate and incubated at 37 °C for 30 min. Human umbilical vein endothelial cells (HUVECs; 1 × 10^4^ cells) were suspended in 100 μl of EBM-2 or endothelial growth medium (EGM; LONZA Inc., Basel, Switzerland), and conditioning medium (CM) of ADSCs, CRP-treated CM of ADSCs or plus VEGF-neutralizing antibody (0.15 μg/ml; R&D Systems Inc.), or EBM-2 plus CRP was added to the solidified Matrigel. The CM was harvested after incubation of the ADSCs in EBM-2 for 24 hours. After incubation on Matrigel at 37 °C in a 5 % CO_2_ chamber, morphological changes were observed under a microscope (Leica, Germany). The five representative fields were photographed. Images were analyzed using Image J software (NIH, Bethesda, MD, USA) to determine the tube lengths.

### In-vivo Matrigel plug assay

The animal experiments were conducted according to the guidelines and ethical standards of the Animal Care and Use Ethics Committees of Sun Yat-Sen University (IACUC-DB-16-070). ADSC (100 μl, 1 × 10^6^ cells) or CRP-treated ADSC (24-hour pretreatment with 25 μg/ml CRP without FBS, 1 × 10^6^ cells) suspensions were mixed with 400 μl of ice-cold growth factor reduced phenol red-free Matrigel (BD Bioscience), and Matrigel containing PBS was used as a negative control. The Matrigel mixture was injected subcutaneously into the dorsal area of male nu/nu mice, 4–5 weeks old. Each experimental condition was performed with three mice. At day 7, the Matrigel implants were removed and then fixed with formalin, and the fixed Matrigel plug was embedded in paraffin to prepare sections for hematoxylin and eosin (H & E).

### Enzyme-linked immunosorbent assay

The assay was performed for the CM using a mouse angiogenesis array kit (R&D Systems, Inc.) according to the manufacturer’s instructions. VEGF-A production was examined by enzyme-linked immunosorbent assay (ELISA) using a commercially available kit (Raybiotech, Atlanta, GA, USA) according to the manufacturer’s instructions.

### Western blot analysis

To prepare the protein extracts, the cells were rinsed twice with ice-cold PBS and harvested. After centrifugation, the cells were resuspended and extracted in lysis buffer (Thermo Fisher Scientific, Inc.) for 30 min on ice. Protein concentrations were assayed using Pierce Coomassie Plus reagent according to the manufacturer’s instructions, and 40 μg of protein was loaded for separation by sodium dodecyl sulfate–polyacrylamide gel electrophoresis (SDS-PAGE). The proteins were then transferred to polyvinylidene difluoride membranes (Immobilon-P; EMD Millipore Corporation, Billerica, MA, USA). The membranes were blocked in Tris-buffered saline containing 5 % bovine serum antigen (BSA) and probed with HIF-1a, tissue inhibitor of metalloproteinase-2 (TIMP-2), VEGF-A, hepatocyte growth factor (HGF), matrix metalloproteinase (MMP)-2, and MMP-9 (all from R&D Systems Inc.) corresponding antibodies. Reacted bands were detected by horseradish peroxidase-conjugated secondary antibodies and enhanced chemiluminescence substrates (PerkinElmer, Boston, MA, USA).

### Quantitative real-time PCR

Total RNA was extracted using Trizol reagent (Thermo Fisher Scientific, Inc.). The synthesis of cDNA was performed on DNaseI-treated total RNA templates (0.5 μg) using an iscript™ cDNA synthesis kit. Gene expression was assessed by quantitative real-time PCR (qRT-PCR) using SYBR Green intercalating dye (Thermo Fisher Scientific, Inc.) and mouse primers. The primer sequences are presented in Additional file 1: Table S1. The comparative threshold cycle method was used to calculate the amplification fold as specified by the manufacturer. The amplified PCR products were separated by gel electrophoresis in a 2 % agarose gel visualized with ethidium bromide. Each sample was replicated at least three times.

### Immunofluorescence staining

The ADSCs were fixed with 4 % paraformaldehyde (PFA) for 10 min, followed by blocking with 5 % BSA in PBS for 60 min at room temperature. The cells were incubated with the following antibodies at room temperature for 1 hour: rabbit anti-CD16/32 (1:200; Abcam Inc.) and rabbit anti-CD64 (1:200; Santa Cruz Biotechnology, Inc., Santa Cruz, CA, USA). Following a wash in PBS, the cells were incubated in goat anti-rabbit secondary antibodies conjugated with FITC (1:200; Thermo Fisher Scientific, Inc.) in PBS for 1 hour at room temperature. DAPI was used for the nuclear stain. The samples were then washed three times, and mounted in mounting medium (Vector Laboratories, Burlington, CA, USA). Images were obtained using an inverted fluorescence microscope.

### Immunoprecipitation

The ADSCs were incubated with CRP (25 μg/ml) for 6 hours and then washed and lysed in 1 ml of RIPA buffer. Cell lysates were precipitated with goat antibodies against CRP (2 μg per 100 g of total protein; Santa Cruz Biotechnology, Inc.) that had been preabsorbed by protein G-Sepharose (Biotool Inc., Houston, TX, USA). Immunoprecipitated proteins were washed in RIPA buffer, subjected to SDS-PAGE, and immunoblotted with specific antibodies against CRP (Santa Cruz Biotechnology, Inc.) or FcgRs (1:200; R&D Systems, Inc.).

### Statistical analysis

The in-vitro data are representative of independent experiments performed in triplicate. The statistical analysis was conducted using SPSS software (SPSS, Inc., Chicago, IL, USA). The statistical significance of the differences among groups was tested using one-way analysis of variance or Student’s *t* test. Error bars are indicative of standard deviation. *p* < 0.05 or *p* < 0.01 was considered significant.

## Results

### Characterization of ADSCs

In this study, we isolated ADSCs from the mouse adipose tissue; the isolated cells were plastic adherent and exhibited spindle-like morphology with a whirlpool-like, colony-forming unit (CFU) of ADSCs (Additional file 1: Figure S1B1, B2). The cells were positive for mesenchymal markers (CD29, CD44, CD90, and SCA-1), and were negative for endothelial (CD105 and CD31), pericyte (CD146), and hematopoietic (TER-119, CD45) markers (Additional file 1: Figure S1A). In addition, the cells were able to differentiate into mesenchymal lineage cells such as adipocytes and osteocytes (Additional file 1: Figure S1B3, B4). Thus, we confirmed that the cells derived from adipose tissue have typical MSC characteristics.

### CRP did not affect ADSC apoptosis or proliferation but increased migration via the PI3K/Akt signaling pathway

**Fig. 1:**
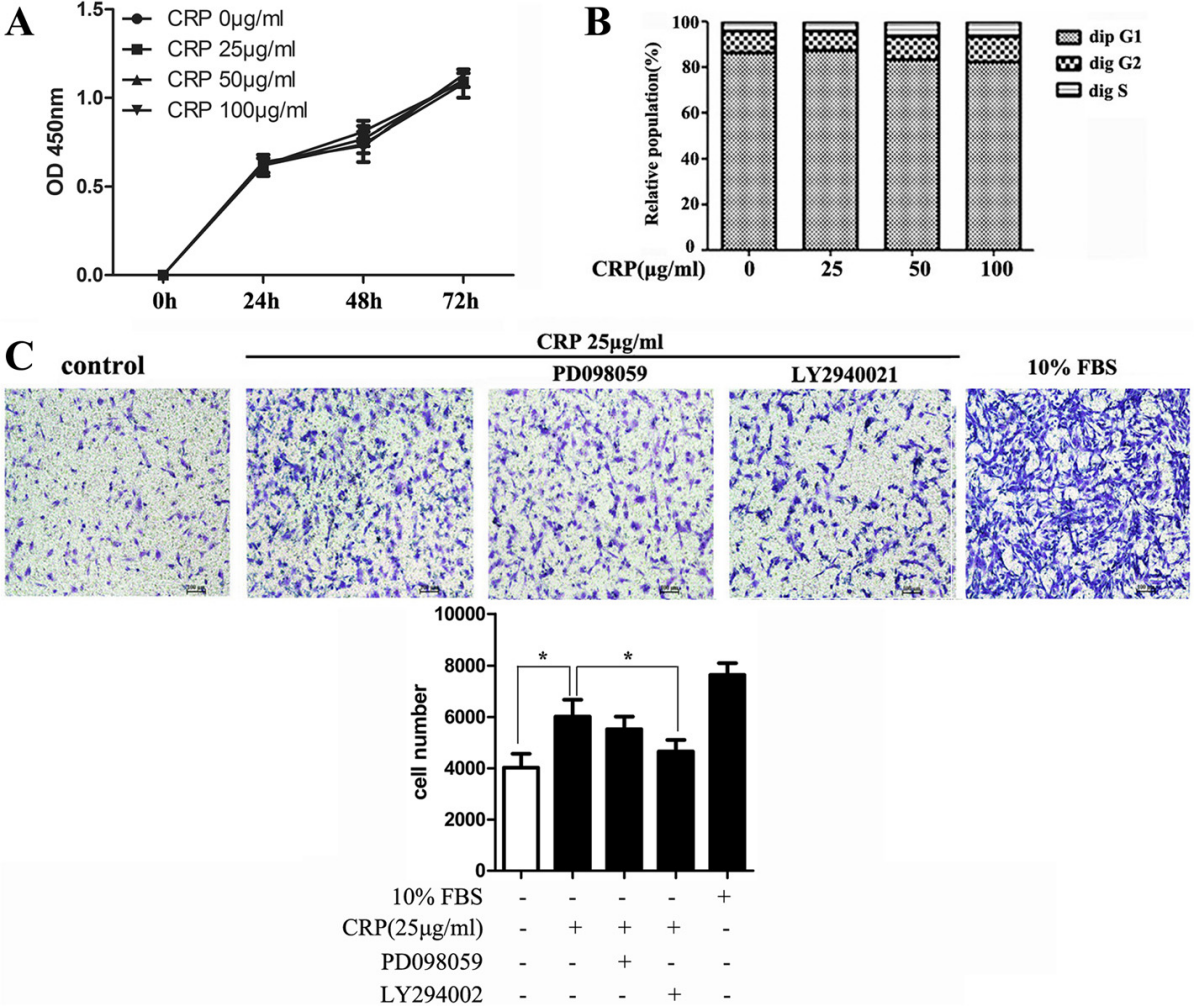
CRP did not affect ADSC apoptosis or proliferation but slightly increased migration via the PI3K/Akt pathway. **a** Proliferation determined by a CCK-8 assay of ADSC cultures 24, 48, and 72 hours after addition of CRP. **b** Distribution of the cell cycle phase of ADSCs in 24-hour cultures with or without CRP treatment. **c** CRP increased the migration of ADSCs and inhibition of Akt (LY294002) significantly inhibited ADSC migration. Data represent mean ± SE (*n* = 3). *Columns*, mean; *error bars*, SEM; *p < 0.05. Results are representative of three independent experiments. *CRP* C-reactive protein, *FBS* fetal bovine serum, *OD* optical density

Several previous studies demonstrated that CRP was associated with cell proliferation and apoptosis in endothelial cells, endothelial progenitor cells, renal tubular epithelial cells, and myeloma cells [20, 21], but this is the first study to investigate the effect of CRP on ADSC proliferation and apoptosis. We found that CRP (0–100 μg/ml) treatment had no significant effects on ADSC proliferation at 24, 48, and 72 hours using a CCK-8 assay (Fig. 1a). CRP treatment slightly increased ADSC migration in a chamber migration assay, which was significantly suppressed by Akt inhibitor (LY294002) treatment (Fig. 1c), indicating that CRP increases ADSC migration via the PI3K/Akt signaling pathway. Our results also showed that CRP treatment did not induce ADSC apoptosis even in the presence of CRP at higher concentrations (100 mg/ml) by Annexin-V binding analysis (data not shown), suggesting that CRP might play a different role in ADSC apoptosis induction.

Because cell cycle mechanisms control stem cell proliferation under normal conditions and stem cells generally remain in the quiescent G_0_ phase in vivo, further investigations were performed to determine whether CRP affected cell cycle regulation by staining with PI and Hoechst 33342. No significant difference was observed between any two groups at different CRP concentrations (0–100 μg/ml) in the distribution of each phase of the cell cycle (Fig. 1b), which is consistent with its function in ADSC proliferation.

### CRP treatment upregulates VEGF-A protein and production in ADSCs

**Fig. 2:**
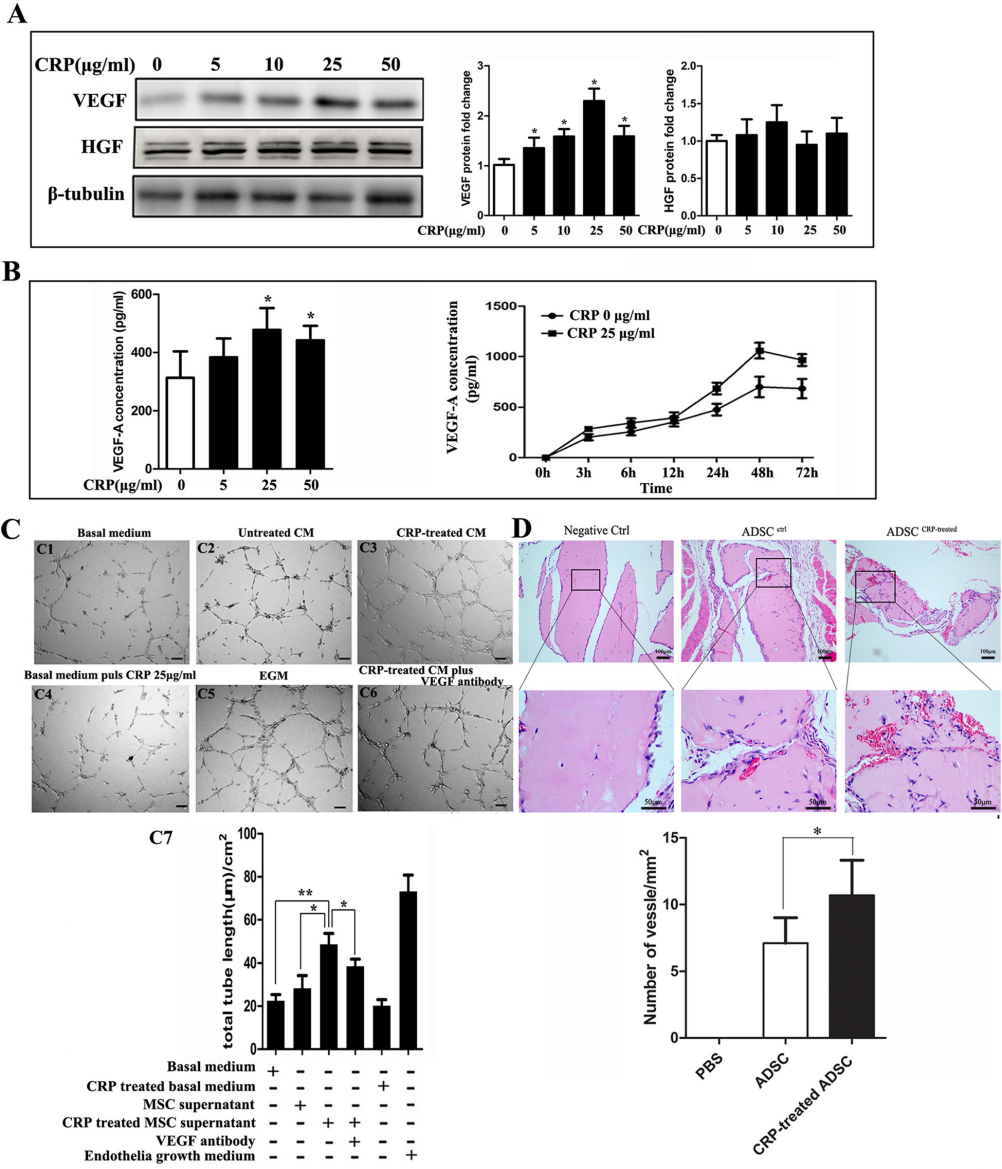
CRP treatment upregulates VEGF protein and production levels and promotes angiogenesis in ADSCs. **a** CRP increased VEGF but not HGF production as assessed by western blotting. Values were normalized to β-tubulin as a control, *p < 0.05 versus control. **b** CRP increased VEGF production dose and time dependently as assessed by ELISA, peak at CRP 25 μg/ml, *p < 0.05 versus control. **c** CRP-induced VEGF increased capillary tube formation in vitro. (*C1*) HUVECs formed tubes in basal medium; (*C2*) culturing of HUVECs in ADSC supernatant induced tube formation; (*C3*) CRP-treated ADSCs conditioned medium (*CM*) enhanced tube formation; (*C4*) CRP-treated basal medium slightly decreased the HUVEC tube formation; (*C5*) HUVECs formed tubes cultured in EGM as positive control; (*C6*) VEGF-neutralizing antibody in CRP-treated ADSC CM prevented HUVEC tube formation; (*C7*) representative histogram of tube length in different medium, *p < 0.05, **p < 0.01. **d** Mice were injected subcutaneously with Matrigel mixed with PBS, ADSCs, and CRP-pretreated ADSCs. At day 7, mice were sacrificed, explanted Matrigel plugs were excised and processed for H & E staining (*upper bars* 100 μm, *lower bars* 50 μm) and light microscope, and microvessel density was quantified by counting vessel structures containing erythrocytes. Representative histogram of tube length in different medium, *p < 0.05; data represent mean ± SEM (*n* = 3). *Columns*, mean; *error bars*, SEM. Results are representative of three independent experiments. *CRP* C-reactive protein, *VEGF* vascular endothelial growth factor, *HGF* hepatocyte growth factor, *ADSC*, adipose-derived stem cell, *EGM* endothelial growth medium, *MSC* mesenchymal stem cell

A recent study demonstrated that CRP preparations might have been contaminated by bacterial endotoxin byproducts and that LPS (200 ng/ml) could promote VEGF-A production in bone marrow-derived mesenchymal stem cells (BMSC) [22]. Accordingly, when we examined the changes of VEGF-A and HGF expression in ADSCs after CRP treatment at different concentrations (0–50 μg/ml) respectively by western blotting and ELISA, we should exclude the effect of LPS potentially present in the CRP preparations. From the results we found that the VEGF-A protein levels were increased after CRP treatment in a dose-dependent manner (Fig. 2a), whereas the HGF protein levels were not. We found that CRP increased VEGF production in ADSCs in a dose-dependent and time-dependent manner (Fig. 2b). VEGF-A protein expression and production was not abrogated by preincubation with polymyxin B (5 μg/ml), which is used to exclude the effect of LPS potentially present in CRP preparations (data not shown). Also CRP 25 μg/ml did not have a significant effect on adipogenic and osteogenic differentiation (Additional file 1: Figure S2).

### CRP-induced VEGF-A upregulation promotes angiogenesis in ADSCs

**Fig. 3:**
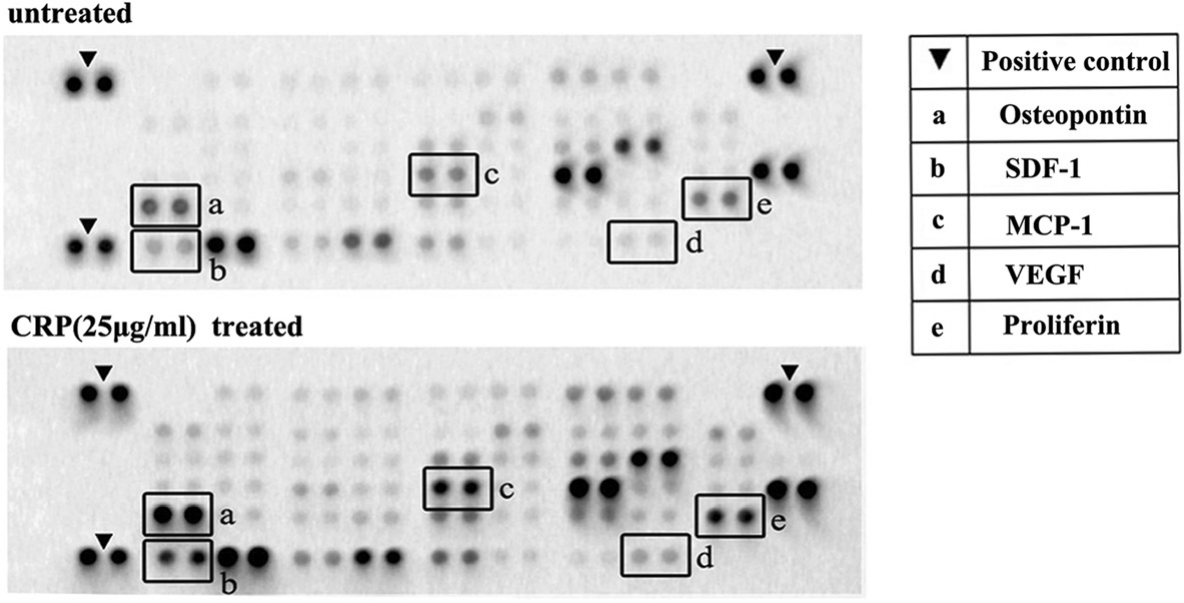
CM with or without added CRP analyzed by antibody-based protein arrays. ADSCs were cultured with 10 % FBS low-glucose DMEM until 80–90 % confluence was reached and then incubated in DMEM for 24 hours. The CM was then collected for protein assays; increased proteins after CRP treatment are indicated with letters. *CRP* C-reactive protein, *VEGF* vascular endothelial growth factor

We then examined the functions of CRP-induced VEGF-A in angiogenesis, and the results demonstrated that HUVECs can form more tubes in ADSC growth medium than in basal medium. In addition, tube length was significantly increased in the CRP-treated ADSC supernatant compared with the normal ADSC supernatant but decreased compared with EGM. This induction effect of CRP can be significantly inhibited by VEGF-neutralizing antibody treatment (Fig. 2c). To further investigate whether CRP promotes ADSC-induced angiogenesis in vivo, the Matrigel plug assay was performed in nu/nu mice. Our results demonstrated that CRP-treated ADSC Matrigel implants showed more functional vessels containing erythrocytes than untreated ADSCs (Fig. 2d). Furthermore, we compared the expression levels of angiogenesis-related proteins in the condition medium (CM) of ADSCs with or without CRP treatment using a commercial antibody assay. Among 55 angiogenesis-related proteins, the expression levels of five were upregulated after CRP treatment—osteopontin, SDF-1, MCP-1, VEGF, and proliferin (Fig. 3)—which are reportedly associated with endothelial cell proliferation, migration, and/or tube formation in vitro.

### CRP promotes MMP-2 proteolytic activity in ADSCs

**Fig. 4:**
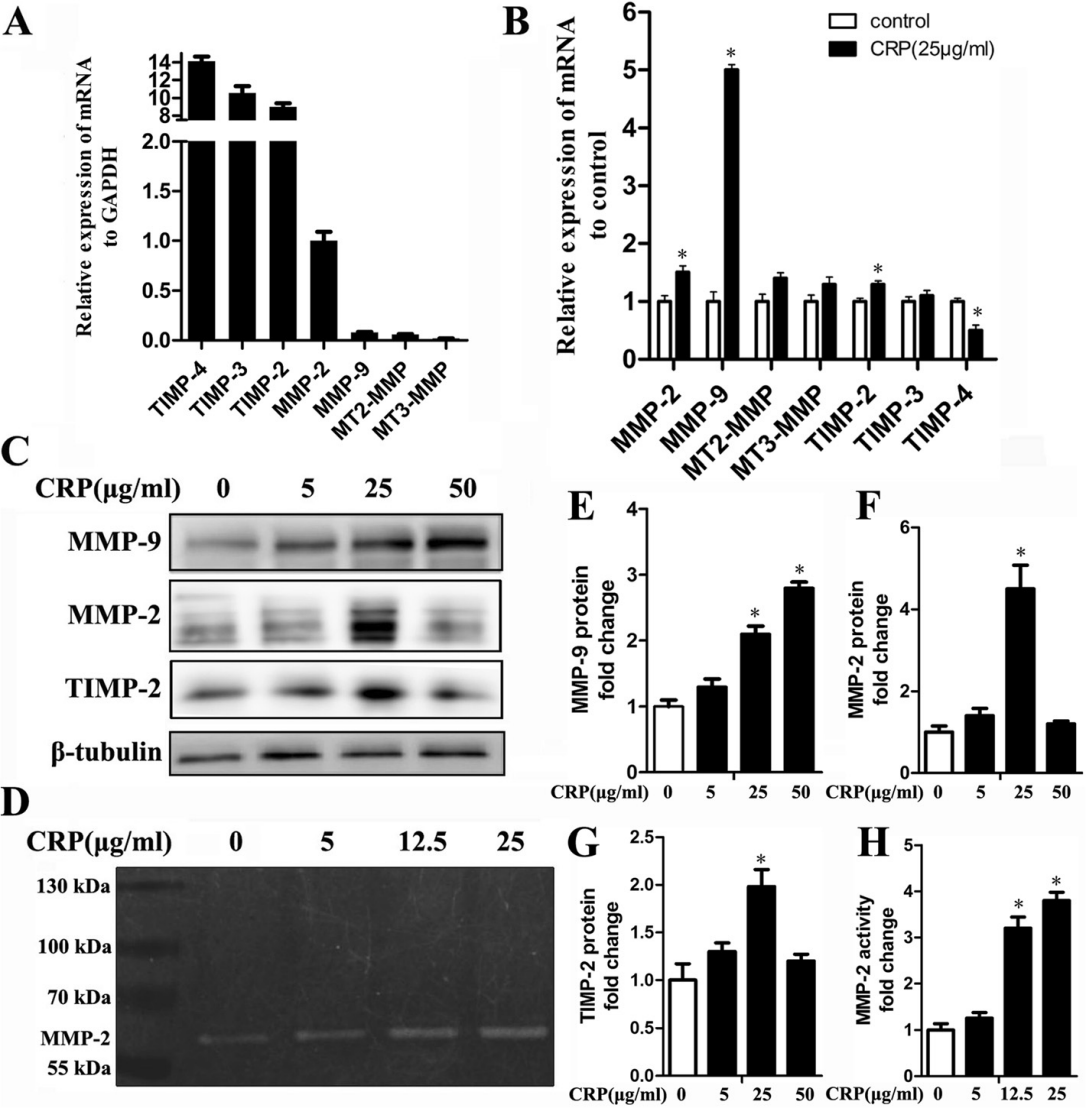
CRP promotes MMP-2 proteolytic activity in ADSCs. **a** RT-PCR analysis of MMP-2, MMP-9, MT2-MMP, MT3-MMP, TIMP-2, TIMP-3, and TIMP-4 gene transcription in ADSCs. Results are mean values ± SD of mRNA expression relative to GAPDH. **b** mRNA expression of MMPs and TIMPs quantified by RT-PCR after 24 hours of incubation with CRP (25 μg/ml) under serum-free conditions. Results given as the fold change in mRNA expression relative to untreated cells set as 1, *p < 0.05, versus control. **c**, **e**, **f, g** CRP significantly increased the gene and protein expression of MMP-2, MMP-9 and TIMP-2. **d** CRP induced MMP-2 proteolytic activity in ADSCs, but MMP-9 proteolytic activity was undetected. *p < 0.05, **p < 0.01 versus control; data represent mean ± SE (*n* = 3). *Columns*, mean; *error bars*, SEM. Results are representative of three independent experiments. *MMP* matrix metalloproteinase, *TIMP* tissue inhibitor of metalloproteinase, *CRP* C-reactive protein

MMP family members and their suppressor TIMP are the dominant factors in transformation of the extracellular matrix (ECM), which is closely related to angiogenesis, so we determined the levels of MMP and TIMP with CRP treatment in ADSCs. Firstly, quantitative determination of mRNA expression in ADSCs by RT-PCR revealed the transcription levels of MMPs and TIMPs compared with that of GAPDH. The expression of MMP-2 mRNA was higher than that of MMP-9, MT2-MMP and MT3-MMP mRNA, whereas MT1-MMP and TIMP-4 mRNA expression was undetectable (Fig. 4a). Moreover, our results showed that CRP treatment can increase MMP-9 mRNA and protein levels in a dose-dependent manner (Fig. 4b, c), but interestingly no MMP-9 activity was found in the supernatant of untreated or CRP-treated ADSCs, which meant MMP-9 activity might be totally suppressed by high-level secretion of TIMP-1 in ADSCs (Fig. 3). Because MMP-2 activity is regulated by MMP-2, TIMP-2, and MT-MMPs in stem cells [23], we next examined the effects of CRP treatment on MMP-2, TIMP-2, and MT-MMP levels. Both qRT-PCR assay and western blot analysis indicated that CRP significantly increased the mRNA and protein expressions of MMP-2 and TIMP-2, but had no effect on the expression of MT2-MMP and MT3-MMP (Fig. 4b, c). We also found that CRP induced MMP-2 proteolytic activity in the supernatant of ADSCs in a dose-dependent manner using gelatin zymography (Fig. 4d). All evidence suggests that MMP-2 proteolytic activity might be further evidence for CRP-mediated angiogenesis in ADSCs.

### CRP upregulates VEGF-A expression by activating HIF-1α via the PI3K/Akt and MAPK/ERK signaling pathways in ADSCs

**Fig. 5:**
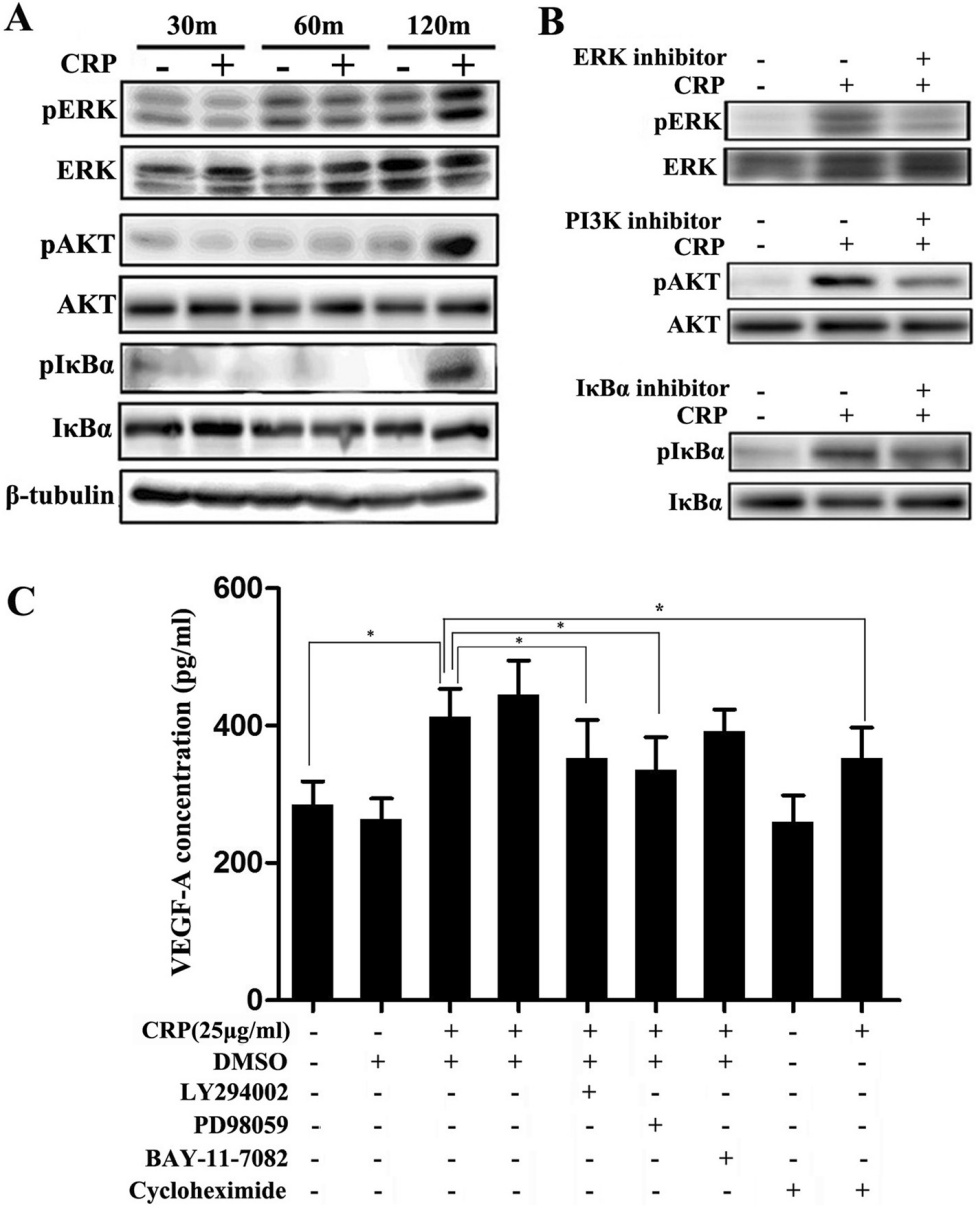
CRP induces phosphorylation of ERK and Akt, and inhibiting both pathways abrogated the increasing of VEGF production. **a, b** CRP induced phosphorylation of ERK, Akt, and NF-kB; the effect peaked at 120 min. Pharmacological inhibitors of MAPK (PD98059), PI3K/AKT (LY294002), and NF-ĸB (BAY-11-7082) inhibited the CRP-mediated increase of phosphorylated kinases. LY249002 (10 μM) for PI3K-specific inhibitor, PD98059 (10 μM) for MAPK inhibitor, BAY-11-7082 (10 μM) for inhibitor for NF-ĸB. Inhibitor concentrations were chosen based on the manufacturer’s recommendations and our preliminary experimental findings. **c** Effects of kinase inhibitors on CRP-induced VEGF production examined by ELISA. Inhibition of the MAPK and PI3K/AKT signaling pathways but not NF-ĸB/IkBα and cycloheximide partly abrogated the increased CRP-induced VEGF production. *Columns*, mean; *bars*, SE. *p < 0.05. The results are representative of three independent experiments. *CRP* C-reactive protein, *VEGF* vascular endothelial growth factor

It was observed that CRP treatment can remarkably induce ERK, Akt, and NF-kB phosphorylation (Fig. 5a). Then we found that the coincubation of cells with CRP and their pharmacological inhibitors of the MAPK pathway (PD98059) or the PI3K/AKT pathway (LY294002) could abrogate the effects of CRP-mediated phosphorylated kinases examined by WB or upregulation of VEGF-A production examined by ELISA in ADSCs, and cycloheximide, a protein expression inhibitor, reduced the increased VEGF-A production induced by CRP (Fig. 5b). However, no detectable difference was observed with or without the coincubation of NK-kB inhibitor (BAY-11-7082) (Fig. 5c).

**Fig. 6:**
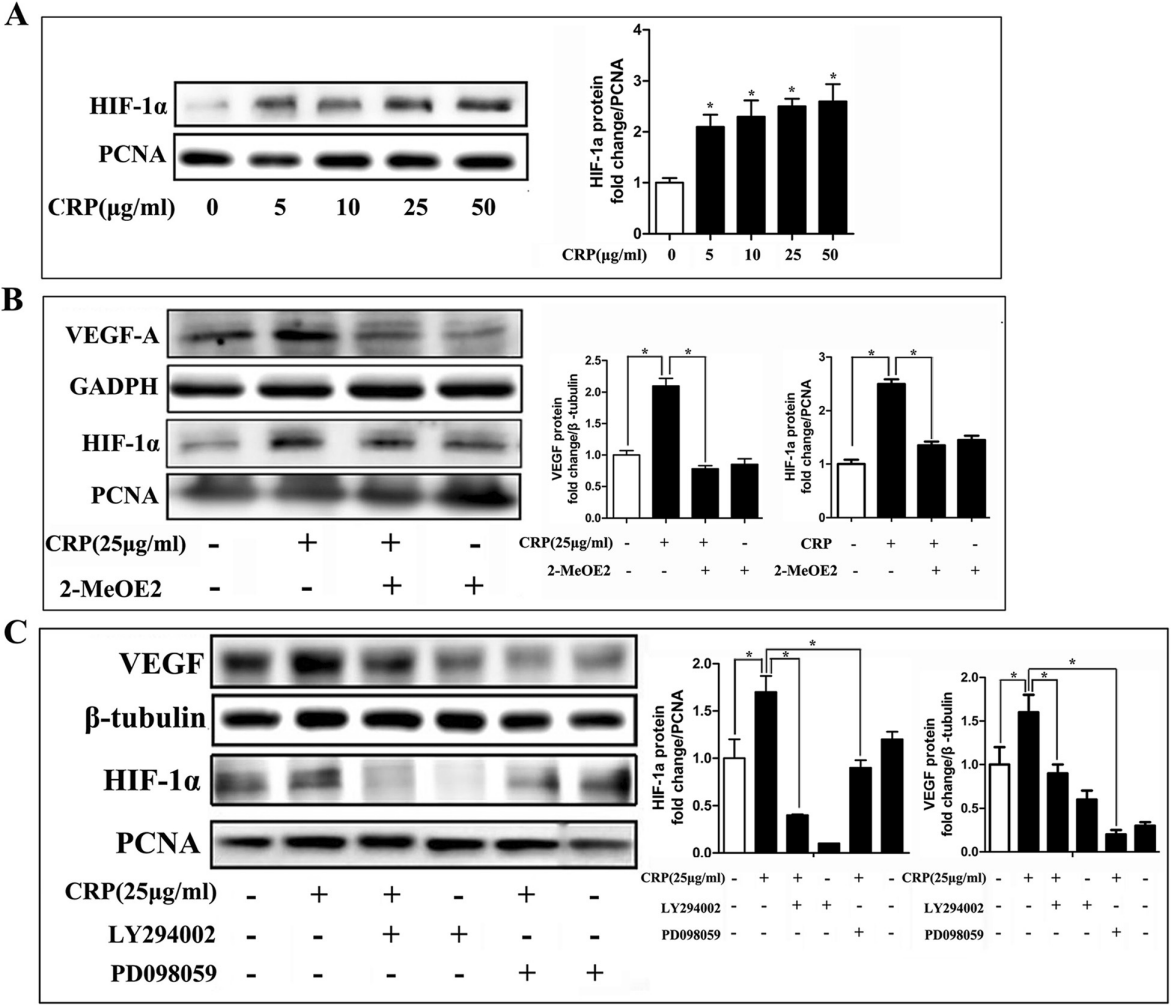
CRP stimulated VEGF expression through HIF-1α, which was linked to activation of the PI3K/AKT1 and MAPK/ERK1/2 pathways. **a** CRP increased HIF-1α production as assessed by western blotting. **b** HIF-1α inhibitor (2-methoxyestradiol, 10 μM) prevented CRP-induced HIF-1α and VEGF protein expression. **c** Effects of kinase inhibitors on CRP-induced HIF-1α and VEGF expression examined by western blotting. MAPK and PI3K signaling pathway inhibition suppressed CRP-induced HIF-1α and VEGF expression. *Columns*, mean; *bars*, SE. *p < 0.05. Results are representative of three independent experiments. *CRP* C-reactive protein, *VEGF*, vascular endothelial growth factor, *HIF-1α* hypoxia inducible factor-1α, *PCNA*, proliferating cell nuclear antigen

Next, we further explored how CRP induced VEGF expression in transcriptional regulation. Regarding the amount of transcription factors such as HIF-1α, AP1, NF-kB, and SP1, HIF-1α is most closely related to VEGF regulation in stem cells. Our results showed that CRP treatment can increase HIF-1α protein expression levels in the nucleus (Fig. 6a). In contrast, further western blot analysis showed that CRP-induced regulation of HIF-1α and VEGF-A protein expression can be suppressed by HIF-1α inhibitor treatment (2-methoxyestradiol, 10 μM) (Fig. 6b), suggesting that CRP may promote HIF-1α to enter the nucleus to induce VEGF expression in ADSCs. Furthermore, our results also showed that ERK and Akt inhibition can suppress CRP-stimulated HIF-1α and VEGF-A protein expression (Fig. 6c), indicating that CRP-induced MAPK/ERK and PI3K/AKT pathway activations were related to HIF-1α activation of VEGF expression in ADSCs.

### CD64 mediated CRP-induced VEGF expression regulation in ADSCs

**Fig. 7:**
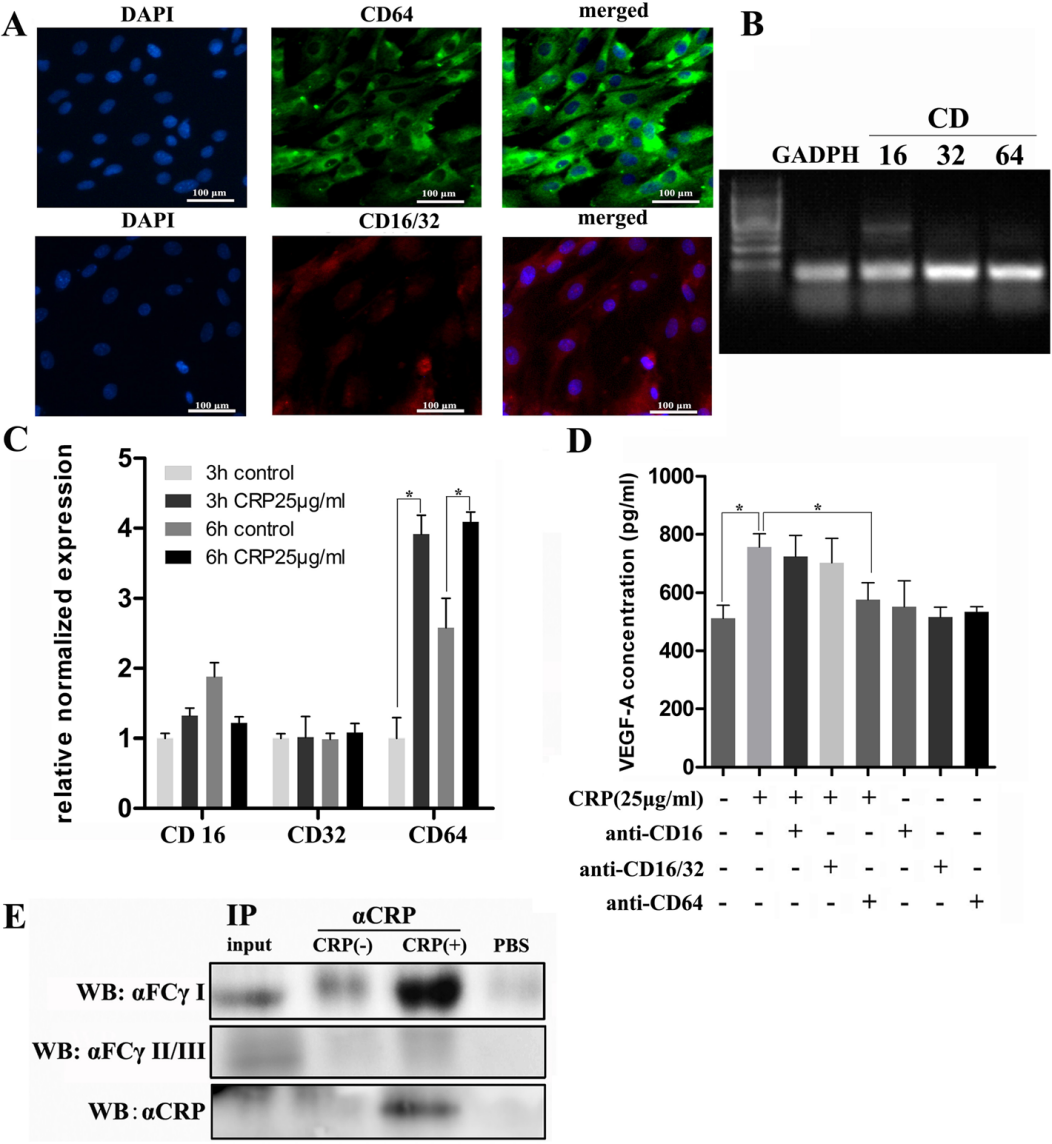
CD64 mediated CRP-induced VEGF expression regulation in ADSCs. **a** Immunofluorescence staining of ADSCs for FcγRIII (CD16), FcγRII (CD32), and FcγRI (CD64) expression in ADSCs. **b** mRNA expression of CD16, CD32, and CD64 detected by PCR amplification and agarose gel electrophoresis. **c** CRP stimulation significantly increased CD64 mRNA expression in ADSCs, whereas no significant difference was detected in CD16 and CD32, *p < 0.05, versus control. **d** Effects of CD16, CD16/32, and CD64 blocking antibody on CRP-stimulated (25 μg/ml) VEGF production in ADSCs in which CD64-neutralizing antibody partly abrogated the effect of CRP. **e** Immunoprecipitation (*IP*) using antibodies against CRP in ADSCs treated with or without CRP (25 μg/ml). Blotting antibodies were against CRP or FcgRI, *p < 0.05. *Columns*, mean; *bars*, SEM. Results are representative of three independent experiments. *CRP* C-reactive protein, *VEGF*, vascular endothelial growth factor, *HIF-1α* hypoxia inducible factor-1α, *WB*, western blot

To understand how CRP acts on ADSCs, further studies were designed to reveal the way in which CRP binds to the ADSC membrane surface receptor. It is well known that CRP shares several functional properties with immunoglobulin G and binds to FcgRs, which are designated Fc gamma RI (also known as CD64), Fc gamma RII (CD32), and Fc gamma RIII (CD16). The findings of RT-PCR and immunofluorescence staining indicated that CD16/32 and CD64 were expressed in ADSCs (Fig. 7a, b). We found that CRP stimulation resulted in increased expression of CD64 mRNA in ADSCs, whereas the CD16 and CD32 mRNA levels showed no significant changes (Fig. 7c). We then examined the roles of CD16, CD32, and CD64 in CRP-regulated VEGF production in ADSCs. Interestingly, specific blocking antibodies for both CD16 and CD16/32 had no significant effects on VEGF-A expression after CRP stimulation in ADSCs, whereas CD64-neutralizing antibody partially abrogated the effect of CRP treatment (Fig. 7d). Furthermore, immunoprecipitation with monoclonal antibodies (mAbs) against CRP was performed to examine whether and which FcgRs bind to CRP on ADSCs. We found that mAbs against CRP can precipitate CD64 but not CD16/32 (Fig. 7e), suggesting that CRP-regulated VEGF expression is CD64 dependent.

## Discussion

Mesenchymal stem cell-induced angiogenesis was found to be regulated mainly by proangiogenic paracrine activity, such as high-level secretions of VEGF, HGF, FGF, and insulin-like growth factor (IGF) [24, 25], which can promote angiogenesis through stimulating endothelial cell maturity, migration, and proliferation. Among these angiogenic factors, VEGF plays a key role in angiogenesis. VEGF secretion levels in ADSCs are low in cultured conditions, our results indicating that CRP can upregulate VEGF expression and production in ADSCs, which significantly increased endothelial cell tube formation in Matrigel, and that CRP pretreated ADSCs formed more functional vessels in vivo. As we know, MMPs play an important role in the formation and maintenance of new capillaries in vivo and in vitro. Previous studies showed that MMP-2 and MMP-9 were involved in coronary artery wall formation in experimental hypercholesterolemia, which coincides with vasa vasorum neovascularization [26]. We found that the addition of CRP significantly increased MMP-2 activity in a dose-dependent manner. In addition, we found CRP had no significant influence on the expression of inflammatory cytokines in ADSCs determined by RT-PCR, such as IL-10, IL-6 and IL-1β (Additional file 1: Figure S3), indicating that CRP-induced angiogenesis in ADSCs may not be driven by inflammatory response. All of this evidence indicates that CRP can increase VEGF production and MMP-2 activity in ADSCs, which triggers endothelial cell activation and accelerates ECM degradation to play a substantial role in subsequent vasa vasorum proliferation.

To further explore the mechanisms of CRP-induced VEGF expression levels, we also examined the activation of HIF-1α, an important transcription factor that regulates VEGF expression via the binding of hypoxia-response element (HRE) sites in the VEGF promoters in stem cells [27, 28] after ADSC CRP treatment. In normoxic conditions, HIF-1 is upregulated by growth factors, cytokines, hormones, and deregulated oncogenes. Our results indicated that CRP can increase HIF-1α protein expression in the nucleus of ADSCs. Moreover, CRP-mediated VEGF and HIF-1α expression changes can be abrogated by HIF-1α inhibitor (2-methoxyestradiol), which can depolymerize the microtubules and inhibit HIF-1α nuclear accumulation and transcriptional activity, indicating that CRP promoted ADSC VEGF production via activating HIF-1α.

**Fig. 8:**
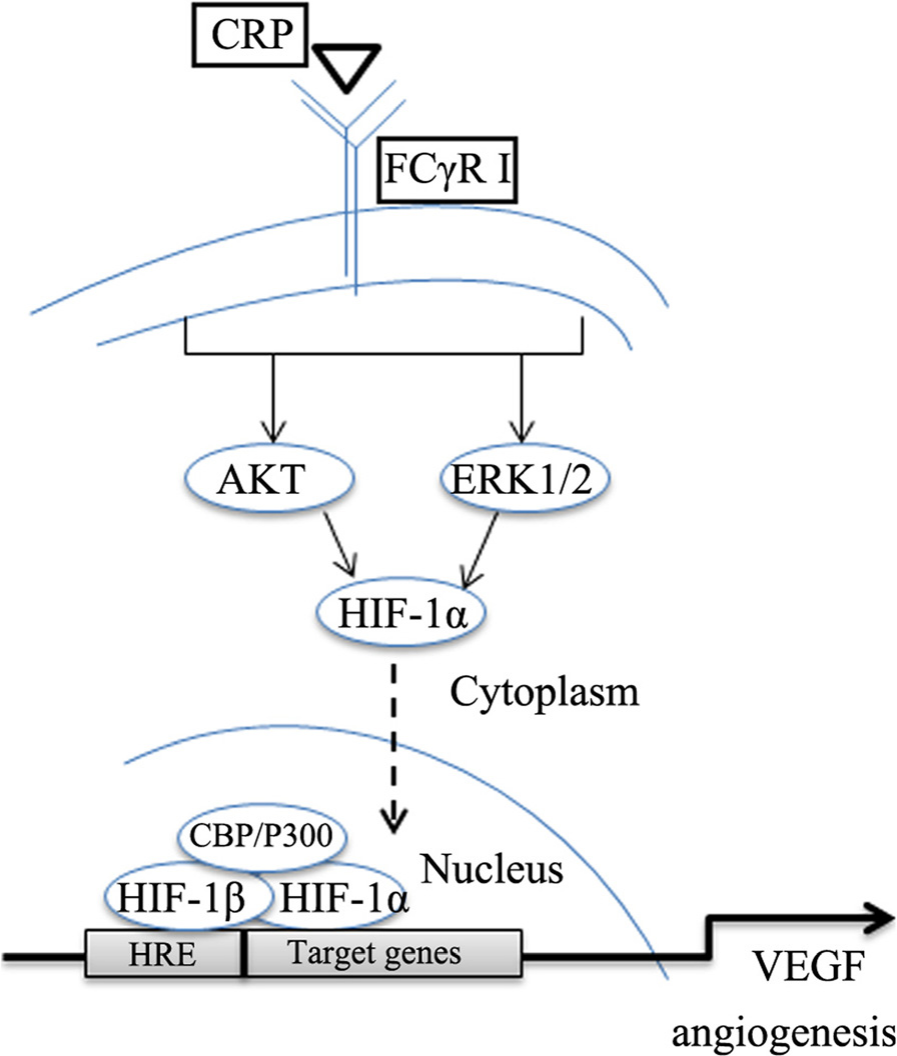
Schematic representation of the molecular mechanism for CRP regulation of VEGF in ADSCs. CRP binds to the membrane surface antigen FCγRI (CD64) on ADSCs and then induces ERK1/2 and Akt phosphorylation, which activates HIF-1α to enter the nucleus and bind to hypoxia-response element sites in the VEGF promoter, stimulating VEGF gene and protein expression. *CRP* C-reactive protein, *VEGF* vascular endothelial growth factor, *HIF-1α*, hypoxia inducible factor-1α, *HRE* hypoxia-response element

Since HIF prolyl hydroxylases (PHDs) regulate HIF-1 during hypoxia conditions, the PI3K/Akt and MAPK/ERK pathways mediate primarily nonhypoxic HIF-1 and VEGF regulation [29]. Firstly, our results showed that CRP can induce the PI3KAkt, MAPK/ERK1/2, and NF-kB/IkBα signaling pathways. As we know, IkBα phosphorylation can activate NF-kB (another VEGF transcription factor) to release it from IkBα complex and bind to HRE sites in VEGF promoters in the nucleus. In the present study, however, only inhibiting the MAPK/ERK and PI3K/Akt pathways, but not the NF-kB pathway, could abrogate CRP-induced VEGF production, which is consistent with the other previous studies [30–32]. Our results further showed that ERK and Akt inhibition can suppress CRP-stimulated HIF-1α and VEGF protein expression, which means that CRP stimulated VEGF expression through HIF-1α in ADSCs was linked to the activation of canonic pathways (PI3K/AKT1 and MAPK/ERK1/2) (see Fig. 8).

CRP can bind to Fcr receptors (FcgRs), which lead to indirect (via classical complement) and direct opsonization (via FcrRs). We found that ADSCs expressed FcgRs, which are also expressed by many kinds of cell types; for example, immune, endothelial, smooth muscle, and tubular epithelial cells [20, 33]. Blocking FcgRI can partially abrogate CRP-stimulated VEGF production, and CRP can bind and precipitate CD64 on ADSCs. Compared with CD16/32, CD64 displays higher affinity to CRP and modulates its actions within immune response cells [34]. This evidence suggested that FcgRI might serve as the major CRP receptor in ADSCs and as a novel therapeutic target for preventing CRP-induced ADSC angiogenesis in the inflammatory condition.

## Conclusion

Our results showed that CRP had no significant effects on ADSC apoptosis and proliferation, whereas it increased ADSC migration via the PI3K/Akt signaling pathway. CRP also significantly stimulated VEGF production, mainly by activating HIF-1α via the CD64/PI3K/Akt and MAPK/ERK signal transduction pathways, and increased MMP-2 activity in ADSCs. Our findings will provide new evidence that CRP could promote growth of the vasa vasorum in atherosclerosis.

## Highlights

1. Previous studies about CRP-induced atherosclerosis mostly focused on the intima and medium, such as the effect of CRP on endothelial cells, SMC, mononuclear cells, and so forth. For the first time, we hypothesized that CRP may play an important role in the development of atherosclerosis by promoting mesenchymal stem cell angiogenesis in periadventitial adipose tissue, and we found that CRP can promote ADSC-induced angiogenesis by stimulating VEGF expression and increasing the activity of MMP-2 in vitro*,* providing new evidence for CRP as a cardiovascular risk factor.
2. For the first time, we found that CRP binds activating CD64 on ADSCs, rather than CD16/32. CD64 may act as a novel therapeutic target for preventing CRP-induced ADSC angiogenesis in the inflammatory condition.

## Abbreviations

ADSC, adipose-derived stem cell; CM, condition medium; CRP, C-reactive protein; EGM, endothelial growth medium; ELISA, enzyme-linked immunosorbent assay; FGF, fibroblast growth factor; HGF, hepatocyte growth factor; HIF-1α, hypoxia inducible factor-1α; HRE, hypoxia-response element; HUVEC, human umbilical vein endothelial cell; MMP, matrix metalloproteinase; PVAT, perivascular adipose tissue; qRT-PCR, quantitative real-time PCR; VEGF, vascular endothelial growth factor

## Additional file

Additional file 1: Table S1. The identification of mADSCs and the effect of CRP on the differentiation potential and inflammation marker in ADSCs. (DOCX 737 kb)
