# Supplementary material for: C-reactive protein can upregulate VEGF expression to promote ADSC-induced angiogenesis by activating HIF-1α via CD64/PI3k/Akt and MAPK/ERK signaling pathways
Source: Stem Cell Res Ther. 2016 Aug 16;7:114. doi: 10.1186/s13287-016-0377-1 (PMC4986362; doi:10.1186/s13287-016-0377-1)
Supplement: Additional file 1: Table S1. — The identification of mADSCs and the effect of CRP on the differentiation potential and inflammation marker in ADSCs. (DOCX 737 kb) [file 13287_2016_377_MOESM1_ESM.docx]

**Table S1 primer sequence**

| **GENE** |  | **sequence** |
| --- | --- | --- |
| VEGF-A | sence | TTACTGCTGTACCTCCACC |
| VEGF-A | antisence | ACAGGACGGCTTGAAGATG |
| HIF-1a | sence | TCTCGGCGAAGCAAAGAGTC |
| HIF-1a | antisence | AGCCATCTAGGGCTTTCAGATAA |
| GADPH | sence | GTGGCAAAGTGGAGATTGTT |
| GADPH | antisence | CTCGCTCCTGGAAGATGG |
| TIMP-1 | sence | CGAGACCACCTTATACCAGCG |
| TIMP-1 | antisence | ATGACTGGGGTGTAGGCGTA |
| TIMP-2 | sence | TCAGAGCCAAAGCAGTGAGC |
| TIMP-2 | antisence | GCCGTGTAGATAAACTCGATGTC |
| TIMP-3 | sence | CTTCTGCAACTCCGACATCGT |
| TIMP-3 | antisence | GGGGCATCTTACTGAAGCCTC |
| TIMP-4 | sence | CACTCGGCTCTAGTGATACGG |
| TIMP-4 | antisence | CTTGGCCTTCTCGAACCCTTT |
| MMP-2 | sence | ACCTGAACACTTTCTATGGCTG |
| MMP-2 | antisence | CTTCCGCATGGTCTCGATG |
| MMP-9 | sence | GCTGACTACGATAAGGACGGC |
| MMP-9 | antisence | AGGAAGACGAAGGGGAAGACG |
| MT1-MMP | sence | ACCCACACACAACGCTCAC |
| MT1-MMP | antisence | GCCTGTCACTTGTAAACCATAGA |
| MT2-MMP | sence | ATGAAGAGACGAAAACGTGGATG |
| MT2-MMP | antisence | TGGAAGACCAATGGTGTGACC |
| MT3-MMP | sence | AGAAGGTTGGATTTCGTGCAT |
| MT3-MMP | antisence | TCCGCAGACTGTAGCACATAA |
| CD16 FcrR III | sence | ACTTTTCAGCCACACAGCCT |
| CD16 FcrR III | antisence | TCCTGATGAGCCGCCTAAAC |
| CD32 FcrR II | sence | GCATTGGGAAAAGCAAGCCA |
| CD32 FcrR II | antisence | AGTCTTGAGTTGGGAAGCTGT |
| CD64 FcrR I | sence | GGTGGATGGGTTCAGGGAAA |
| CD64 FcrR I | antisence | GGGAGGGTGCATGAAGAGAG |


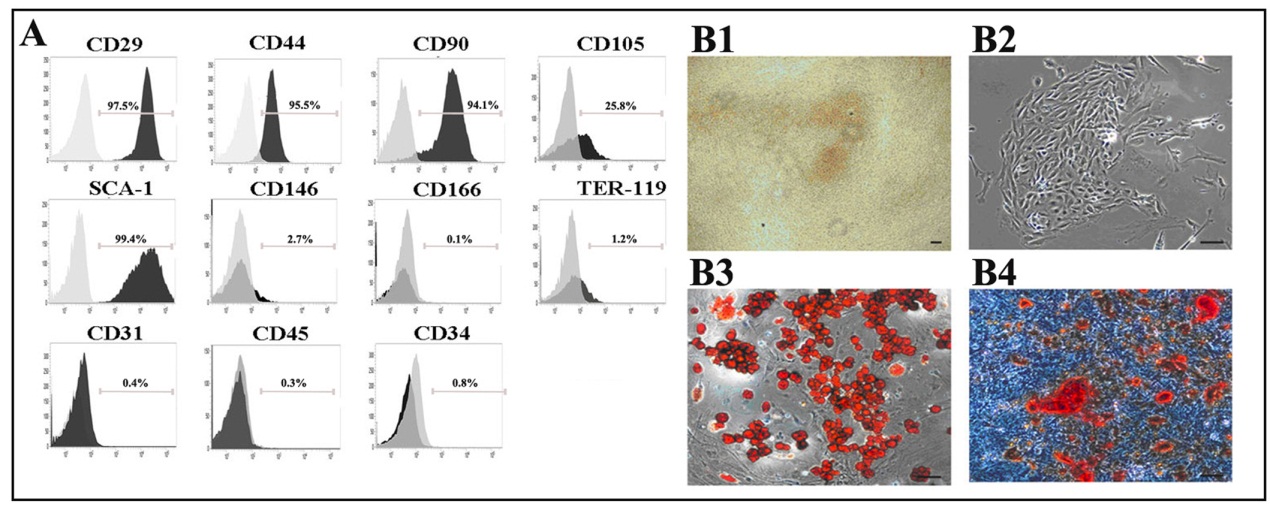


**Figure S1 Characterization of TER119-/CD31-/CD45-mADSCs. (A)** Immunophenotypic characterization of mouse adipose deprived stem cells (mADSCs) by flow cytometry. Representative single-parameter diagrams showing the expression of mesenchymal (CD90/Thy-1, CD44/Pgp-1, CD29/b1-integrin, SCA-1, CD166/ALCAM, CD34), endothelial (CD105/endoglin, CD31/PECAM-1), pericyte marker(CD146/MUC18), hematopoietic(TER-119,CD45/Lyt-4). **(B1)** Spindle-like cells with a whirlpool-like. **(B2)** Colony Forming Unit(CFU). **(B3)** Oil Red O staining of intracytoplasmic lipid-rich droplets in the ASCs after adipogenic induction for 21 days. **(B4)** Alizarin red staining of calcium phosphate precipitates in ASCs at day 21 after osteogenic induction.


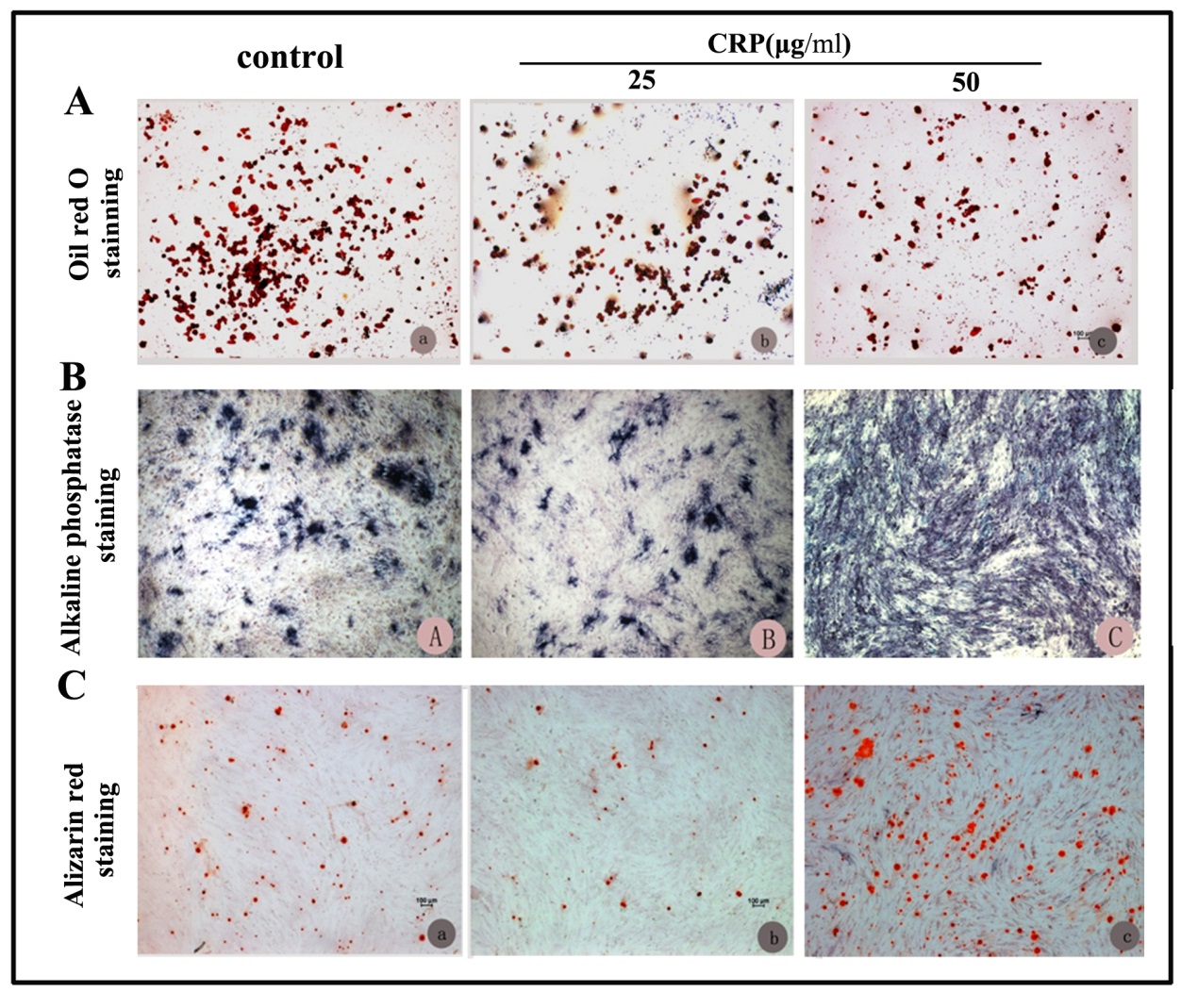


**Figure S2 the effect of CRP on adipogenic and ostogenic differenation of ADSCs (A)** CRP 25ug/ml did not affect adipogenic differentiation of ADSCs, but CRP 50ug/ml significantly suppressed adipogenesis of ADSCs. The quantity of lipids in the ADSCs was determined by measuring the absorbance of the oil red O extracted from the cells. **(B)&(C)** CRP 25ug/ml also did not affect ostogenic differentiation, but 50ug/ml significantly enhances osteoblast differentiation. Osteoblast differentiation was analyzed by alkaline phosphatase staining and alizarin red S staining.


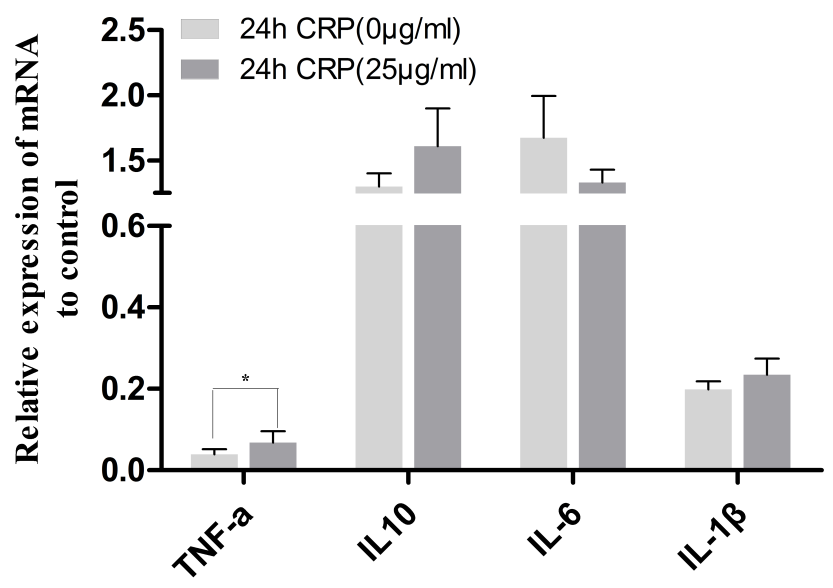


**Figure S3 the effect of CRP on the expression of inflammatory markers in ADSCs.** CRP had no significant influence on the expression of IL-6, IL-10 and IL-1β of in ADSCs examined by RT-PCR, except for TNF-α. *P < 0.05, versus control; data represent mean ± SE (n = 2). Columns, mean; error bars, SEM. The results are representative of two independent experiments. RT-PCR, real-time polymerase chain reaction;
